# Dental plaque bacteria with reduced susceptibility to chlorhexidine are multidrug resistant

**DOI:** 10.1186/s12866-016-0833-1

**Published:** 2016-09-15

**Authors:** Hafiz Ghulam Murtaza Saleem, Christine Ann Seers, Anjum Nasim Sabri, Eric Charles Reynolds

**Affiliations:** 1 Department of Microbiology and Molecular Genetics, University of the Punjab Quaid-e–Azam Campus, Lahore, Pakistan; 2 Oral Health Cooperative Research Centre, Melbourne Dental School, and The Bio21 Institute, The University of Melbourne, Melbourne, VIC 3010 Australia

**Keywords:** Chlorhexidine, *Chryseobacterium culicis*, *Chyseobacterium indologenes*, Antimicrobial resistance

## Abstract

**Background:**

Chlorhexidine (CHX) is used in oral care products to help control dental plaque. In this study dental plaque bacteria were grown on media containing 2 μg/ml chlorhexidine gluconate to screen for bacteria with reduced CHX susceptibility. The isolates were characterized by 16S rRNA gene sequencing and antibiotic resistance profiles were determined using the disc diffusion method.

**Results:**

The isolates were variably resistant to multiple drugs including ampicillin, kanamycin, gentamicin and tetracycline. Two species, *Chryseobacterium culicis* and *Chryseobacterium indologenes* were able to grow planktonically and form biofilms in the presence of 32 μg/ml CHX. In the CHX and multidrug resistant *C. indologenes* we demonstrated a 19-fold up-regulation of expression of the HlyD-like periplasmic adaptor protein of a tripartite efflux pump upon exposure to 16 μg/ml CHX suggesting that multidrug resistance may be mediated by this system. Exposure of biofilms of these resistant species to undiluted commercial CHX mouthwash for intervals from 5 to 60 s indicated that the mouthwash was unlikely to eliminate them from dental plaque in vivo.

**Conclusions:**

The study highlights the requirement for increased vigilance of the presence of multidrug resistant bacteria in dental plaque and raises a potential risk of long-term use of oral care products containing antimicrobial agents for the control of dental plaque.

## Background

The oral diseases (dental caries and periodontal diseases) are a major public health problem and are amongst the most prevalent diseases of mankind [1]. The main cause of these diseases is the complex microbiota established as dental plaque, a complex microbial biofilm [2] containing over 750 different bacterial species [3].

Dental plaque biofilms provide a resistant environment for bacteria due to their stable structures being uniquely accreted to non-shedding surfaces. Bacterial biofilms show increased tolerance to antibiotics and antiseptics and resist phagocytosis as well as other components of host defense, which can ultimately lead to chronic infections [4, 5]. Regular oral hygiene is vital for oral health. Brushing and flossing are considered the gold standard oral hygiene procedures to help control dental plaque. However, even though much emphasis is placed on the mechanical methods of oral hygiene, oral care products such as toothpastes and mouthwashes containing antimicrobial agents are actively promoted by manufacturers as a means to assist in the control of plaque and gingivitis [6–10]. A range of antibacterial (antiseptic) agents such as chlorhexidine (CHX), triclosan, essential oils and metal salts Sn^II^, Zn^II^ are used in commercial oral care products to help control dental plaque and halitosis [11, 12]. CHX exhibits broad spectrum activity against both Gram-positive and Gram-negative bacteria, yeast, dermatophytes and lipophilic viruses [13] and is considered stable, safe and effective in helping to reduce plaque and gingivitis [14–16].

CHX has been used as an antiseptic for many decades. Although uncommon, some resistance has emerged in clinical isolates with reduced susceptibility to CHX such as multiresistant *Staphylococcus aureus* [17] and other species [18, 19]. The major factor for the progression of bacterial resistance to antiseptic agents has been suggested to be the selective pressure created by over-use of antibiotics [20]. However, the long term use of oral care (mouthwash and toothpaste) antimicrobials (chlorhexidine and triclosan) may also be a contributing factor to the development of multidrug resistance [21, 22]. The widespread, uncontrolled use of antibiotics and antiseptics may lead to the ultimate selection of multidrug resistance strains that can occupy a niche in dental plaque after competition with the susceptible normal flora. The niche can then act as a source for dissemination of the multidrug resistant strain and establishment of a life-threatening infection in a compromised host [23–26].

This study sought to identify bacteria less susceptible to CHX in dental plaque and then to determine the antibiotic resistance characteristics of these CHX-resistant plaque isolates.

## Results

### Identification of dental plaque isolates

Dental plaque from 5 individuals was isolated, suspended in PBS, serially diluted and plated on nutrient agar (NA). Bacteria with variant morphology were selected and passaged on to NA supplemented with CHX at 2 μg/ml. Six colonies with variant morphologies were selected for further analysis. Genomic DNA was extracted from the isolates and the 16S rRNA gene sequenced. The sequences were used for species identification using BLAST similarity search at the National Center for Biotechnology Information (http://blast.ncbi.nlm.nih.gov/Blast.cgi). Maximum identities were found to *Chryseobacterium culicis*, *Chryseobacterium indologenes*, *Acinetobacter johnsonii*, *Enterobacter ludwigii*, *Pseudomonas stutzeri* and *Streptococcus salivarius* (Table 1).

**Table 1:** Name and accession number of dental plaque bacteria exhibiting chlorhexidine resistance

Species	16S Identity (%)	GenBank Accession no.
*Chryseobacterium culicis*	98	KR002422
*Chryseobacterium indologenes*	98	KR002424
*Acinetobacter johnsonii*	99	KR002423
*Enterobacter ludwigii*	99	KR002425
*Pseudomonas stutzeri*	99	KC817808
*Streptococcus salivarius*	99	KC817807

### Dental bacteria display variable antibiotic resistance profiles

The six isolates were tested for their susceptibility to antimicrobial compounds representing β-lactams (ampicillin), aminoglycosides (gentamicin and kanamycin), tetracyclines (tetracycline) and macrolides (erythromycin) plus chloramphenicol and the non-ribosomal peptide vancomycin using disc diffusion. The bacteria showed variation in their resistance profiles (Table 2) with *Chryseobacterium* species found to be the most resistant to the drugs including ampicillin, kanamycin, gentamicin, and tetracycline whereas they were susceptible to erythromycin and vancomycin with ≥18 mm zones of inhibition. *S. salivarius* was susceptible to vancomycin, ampicillin, and chloramphenicol but resistant to kanamycin and tetracycline. *A. johnsonii* was sensitive to kanamycin, tetracycline and chloramphenicol but was resistant to vancomycin, ampicillin and erythromycin. *E. ludwigii* was erythromycin sensitive (17 mm zone of inhibition) but gentamicin, kanamycin and tetracycline resistant. *P. stutzeri* had intermediate resistance against four different drugs, susceptibility to chloramphenicol and resistance to kanamycin and tetracycline (Table 2). Therefore, when CHX resistance was identified the species were also resistant to a range of antibiotics.

**Table 2:** Antibiotic profile of plaque microorganisms

Organisms	Antibiotics Zones of inhibition (mm)						
	Vm	Ap	Km	Gm	Tc	Em	Cm
*C. culicis*	18	0	4	11	10	18	11
*C. indologenes*	19	6	4	11	7	22	10
*A. johnsonii*	4	10	17	16	17	10	26
*E. ludwigii*	14	5	8	13	11	17	16
*S. salivarius*	23	32	10	15	11	14	28
*P. stutzeri*	14	16	12	15	13	14	23
*E. coli* (*control*)	1	17	21	20	22	17	25
*S. aureus*( *control*)	17	26	20	21	28	18	20

*Vm* vancomycin, *Ap* ampicillin, *Km* kanamycin, *Gm* gentamicin, *Tc* tetracycline, *Em* erythromycin, *Cm* chloramphenicol

Susceptible: ≥17 mm; Intermediate: 14–16 mm; Resistant: ≤ 13 mm

*E. coli*, *Escherichia coli* ATCC 25922; *S. aureus*, *Staphylococcus aureus* ATCC 25923

### Isolates demonstrated biofilm formation in the presence of CHX

**Fig. 1:**
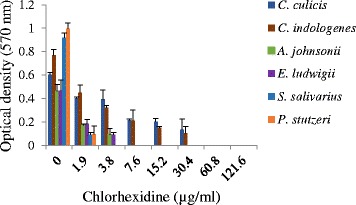
The biofilm forming ability of dental isolates in the presence and absence of chlorhexidine. Biofilms were stained with crystal violet, destained and released crystal violet measured

The presence of bacteria in plaque requires the capacity to form biofilm. CHX insult may affect this phenotype. We therefore tested the capacity of these species to form biofilm in vitro in the presence of CHX. *C. culicis* and *C. indologenes* could form biofilms in the presence of 32 μg/ml of CHX. *A. johnsonii* and *E. ludwigii* biofilms were produced when grown in up to 3.8 μg/ml of CHX, whilst biofilms of the other species did not form in CHX above 1.9 μg/ml concentration (Fig. 1).

### Exhibition of increased MIC by plaque microorganisms

**Fig. 2:**
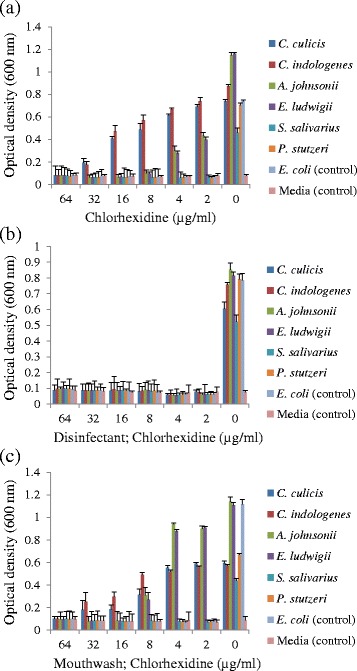
MIC of chlorhexidine against oral isolates when presented as (**a**), chlorhexidine gluconate (Sigma); (**b**), disinfectant (Dentalife); (**c**), mouthwash (Savacol)

To gauge how the resistance to CHX supplied as a pure chemical related to the efficacy of CHX when presented in commercial CHX-containing antiseptic and mouthwash, cultures were also grown in media containing serial dilutions of a CHX-containing disinfectant and mouthwash (Savacol) (Fig. 2). No species was able to grow in the presence of the disinfectant at any of the dilutions tested. Chlorhexidine gluconate and the commercial mouthwash had activity against *A. johnsonii* and *E. ludwigii* at 4 μg/ml or 8 μg/ml effective CHX respectively. The growth of *S. salivarius* and *P. stutzeri* were completely inhibited by CHX at ≥2 μg/ml from all three sources. The MIC for *Chryseobacterium* sp. was 32 μg/ml effective CHX from the commercial mouthwash, equivalent to CHX as pure chlorhexidine gluconate (Fig. 2).

### Bacteria showed viability after exposure to CHX mouthwash

**Fig. 3:**
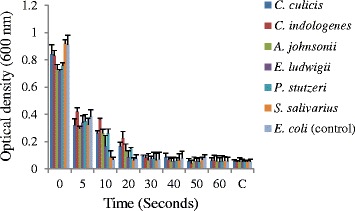
Recovery of dental isolates following biofilm exposure to the commercial CHX mouthwash (Savacol) for different time intervals

To determine if an oral hygiene regime using a CHX containing mouthwash would be effective against these species, with a reduced susceptibility to CHX, the bacteria were grown as a biofilm in microtiter plates and exposed to the commercial mouthwash for different time intervals. The exposed biofilms in the wells were washed and surviving bacteria grown in fresh media overnight. The cells in the planktonic phase were aspirated to a fresh plate and the optical density determined. The bacteria biofilms exhibited different patterns of viability after exposure. *C. culicis*, *C. indologenes*, *A. johnsonii* and *P. stutzeri* showed some tolerance and could grow after 20 s exposure to the undiluted mouthwash whereas after 30 s their viability was diminished (Fig. 3). *E. ludwigii* and *S. salivarius* could grow after 10 s exposure whilst the control, sensitive species *E. coli* only grew following the 5 s exposure interval.

### Mechanism of CHX resistance in *C. indologenes*

*C. indologenes* is known to be a lethal opportunistic pathogen, with inherent antimicrobial resistances [27]. The determination that it is also resistant to antiseptics is of concern and determination of the mechanism warrants investigation. CHX resistance, as with resistance to other antimicrobials can be mediated by efflux [28, 29]. Bioinformatic analysis of the *C. indologenes* NBRC 14944 genome (GenBank RefSeq assembly accession: GCF_000520835.1) indicated that it encodes numerous proteins predicted to be efflux pumps and transporters. WP_034734960 (Ref Seq accession, coded by locus CIN01S_RS05745) is a putative AcrB/HlyD-like periplasmic adaptor protein component of a tripartite efflux pump complex. To test if this may be involved in CHX efflux we exposed *C. indologenes* to 16 μg/ml CHX and compared the gene expression to that in cells not exposed to CHX. Comparison of Cq determined from real-time qPCR from four separate experiments consistently showed an approximately 4 Cq difference between test and control samples. This indicated a 19.3 ± 1.18-fold up-regulation of this gene (Table 3) following CHX exposure.

**Table 3:** Comparative Cq for CIN01S_RS05745 transcription with and without CHX exposure

Experiment	Cq, no chlorhexidine	Cq, 16 μg/ml chlorhexidine	Cq Difference	Fold^b^
1	21.35277 ± 0.66	17.12959 ± 0.07	4.23	18.77
2	22.44871 ± 0.17	17.99964 ± 0.01	4.45	21.86
3	25.59883 ± 0.23	21.29023 ± 0.02	4.30	19.70
4	29.26634 ± 0.19	25.18519 ± 0.27	4.08	16.91
			Mean^a^	19.30 ± 1.18

^a^with standard error

^b^calculated as 2^(mean)^

## Discussion

Mouthwash and toothpaste containing antimicrobials (CHX, triclosan, essential oils) are used to help control the growth of dental plaque [30]. Extensive use of antimicrobials (antiseptics and antibiotics) may result in the selection of resistance bacterial strains which may cause life-threatening infection or superinfection in compromised individuals [21, 22, 31]. In the current study dental supragingival plaque was isolated from 5 healthy individuals and 6 species were isolated that could grow in the presence of 2 μg/ml of CHX. When examined further, these species also exhibited resistance to a variety of antibiotics. Two isolates, *C. culicis* and *C. indologenes*, exhibited resistance to five of the different drugs tested: ampicillin, chloramphenicol, kanamycin, tetracycline and gentamicin, consistent with reports that constitutive multidrug resistance is common in C*hryseobacterium* species [32]. The MIC values for CHX exhibited by the C*hryseobacterium* species reported in this current paper are amongst the highest reported [19]. Knowledge of *C. culicis* is scant, being limited to the phenotypic characteristics determined during its classification [33]. *C. indologenes* (formerly known as *Flavobacterium indologenes*, or *Flavobacterium aureum*) on the other hand, is known to be distributed in the environment but is capable of being a lethal nosocomial pathogen, particularly of infants [34] and persons who are immunocompromised or with underlying morbidities [35]. The *E. ludwigii* isolate had resistance against four drugs so it was the second most resistant bacterium. *E. ludwigii* is a recently recognized species [36] and is a member of what is referred to as the *Enterobacter cloacae* complex, which is six *Enterobacter* species clustered due to their DNA similarity (61–67 %) [37]. The other cluster species are *E. cloacae*, *Enterobacter asburiae*, *Enterobacter hormaechei*, *Enterobacter kobei* and *Enterobacter nimipressuralis*. These species have emerged as nosocomial pathogens, however, unlike this dental isolate most isolates of the *E. cloacae* complex are susceptible to chloramphenicol, aminoglycosides and tetracyclines, while they are intrinsically resistant to ampicillin [37]. The *A. johnsonii* isolate showed resistance against vancomycin, ampicillin and erythromycin. A previous study has reported multidrug resistant *Acinetobacter* species as emerging pathogens, most frequently in association with pulmonary infections [38]. A relationship between oral health and respiratory conditions has been noted [39, 40]. Also, correlation between oral care and reduction in pulmonary infections in the elderly and ventilator-associated pneumonia demonstrated [41, 42]. Furthermore, deep neck infections are more associated with species of dental origin than pharyngotonsillar species [43].

The rate of integration of these species in the dental plaque of populations is at present unknown and it cannot be ruled out that they were transient components of the plaque microbiome of the individuals. Transient or otherwise, a more in depth investigation of the prevalence of these species in plaque is particularly important considering that more than one of these species has potential association with harmful infections in vulnerable individuals. The presence of multidrug resistant species in the oral cavity may increase the risk of patients acquiring infections and superinfections that are difficult to control and also suggests that the oral sources of infectious agents should be a consideration in patient care.

Our study indicated potential ineffectiveness of tetracycline and kanamycin for antimicrobial treatment of infections in the cohort as five of the dental isolates were resistant to these drugs. Similarly, ampicillin was ineffective against the plaque bacteria except for *S. salivarius* and *P. stutzeri* which showed some sensitivity. Our results are similar to Maripandi et al., who found multi-resistance of dental bacteria to chloramphenicol, vancomycin, penicillin and streptomycin [44]. The selective nature of the chlorhexidine-containing media used here precluded determination of the total percentage of species the chlorhexidine resistant organisms represent. Comparison of a total viability count could enable this to be estimated in future studies if desired.

Co-exposure to antibiotics and antiseptics may contribute to the selection of multidrug resistant strains [21, 22, 45]. Multidrug resistance phenotypes can be achieved by microorganisms by a variety of mechanisms, with a common mechanism being efflux of the drug from the cell. Efflux removes those compounds that breach the membrane barriers *via* pumps located in the cytoplasmic membrane of both Gram-positive and Gram-negative bacteria. Efflux pumps can utilize the energy of ATP binding and hydrolysis, or transmembrane proton or Na^+^ potentials to facilitate transport of antimicrobials out of the cytoplasm [46]. Gram-negative species also have tripartite efflux pumps that span the inner and outer membranes to enable substrate efflux from the cytoplasm or periplasm to the extracellular environment [28, 46]. These tripartite systems have an inner membrane pump, a periplasm adaptor protein and an outer membrane channel. The most well studied and most significant clinically are members of the RND (resistance nodulation division) family [46].

Efflux pumps can have specific substrates however the association of multidrug resistance with individual efflux pumps has been established. For example, the MexAB-OprM system in *P. aeruginosa* confers intrinsic resistance to numerous classes of compounds, including β-lactams, macrolides, lincosamides, tetracyclines, quinolones, chloramphenicol, trimethoprim, sulfamethazole and nofloxacin [46]. The *E. coli* AcrAB-TolC system confers resistance to these compound as well as antiseptics [46]. Multidrug resistant *Acinetobacter baumanii* is also resistant to chlorhexidine, effluxing this compound via the AdeAB system which also exports numerous other compounds [47]. Another example is the AceI transporter of the newly recognized Proteobacterial Chlorhexidine Efflux (PCE) protein family [48].

We demonstrated a 19-fold up-regulation of the gene CIN01S_RS05745 encoding the HlyD-like periplasmic adaptor protein of a tripartite efflux pump of *C. indologenes* upon exposure to 16 μg/ml CHX. This responsiveness to CHX stress by *C. indologenes* suggests that tolerance to CHX may be mediated by efflux involving this system. The promiscuity of efflux pumps with respect to substrates can mean that development of resistance to one antimicrobial can have a pleiotropic effect on the resistance profile of a species. Chlorhexidine resistance may emerge as a co-effect due to over-exposure to other antimicrobials. However, it is also possible that longer term exposure to antimicrobials in oral care products has led to the development of resistance and this could have broad implications for development of resistance to other antimicrobials, including antibiotics.

The CHX-containing antiseptic (Dentalife) had efficient growth inhibiting effect against all the oral isolates in contrast to the commercial mouthwash where *Chryseobacterium* species were able to grow up to an MIC value of 32 μg/ml and *A. johnsonii* showed resistance against CHX with an MIC 4 μg/ml. The greater effectiveness of Dentalife is likely attributable to other additives in the disinfectant acting in synergy with the chlorhexidine. An oral formulation (mouthwash) is restricted by the need to be safe, a low irritant of the oral mucosa and palatable, restraints that do not apply to a surface antiseptic.

Bacteria in biofilms such as dental plaque are more resistant to CHX due to the poor penetration of the antimicrobial [49]. Furthermore, plaque inhibition by a CHX mouthwash is dependent upon the dose and time of exposure [50]. Herein we show that dental plaque species resistant to multiple antimicrobials are also able to resist short term exposure to CHX. Previous studies have reported that in vivo dental plaque regrowth inhibition requires mouth rinsing twice daily for 60 s with 10 ml of a 0.2 % solution of CHX [16] and that even after a 60 s exposure to 0.2 % solution of CHX, a considerable number of biofilm bacteria remained viable [51]. Hence this suggests that the commercial CHX mouthwash would not be able to eliminate the multidrug resistant dental plaque bacteria. It has been shown in the laboratory that *Streptococcus sanguis* under CHX pressure can develop reduced susceptibility to CHX which is transmissible [52]. This may raise concerns about the long-term use of these products in terms of developing multidrug resistance in dental plaque bacteria. Although studies involving use of CHX-containing products have shown no long lasting effect on the prevalence of species with lowered susceptibility to CHX post cessation of CHX use [53].

## Conclusions

We have demonstrated the existence of dental plaque bacteria resistant to multiple drugs as well as the antiseptic CHX. The study highlights the need for increased awareness of the presence of multidrug resistant bacteria in dental plaque and if long-term use of antimicrobial oral care products is contributing to the emergence of multidrug resistant dental plaque bacteria, then the risk/benefit of using these products may need to be re-evaluated.

## Methods

### Isolation and identification of plaque bacteria

Human dental plaque samples were collected from five healthy individuals from the Dental Hospital, Lahore, Pakistan with informed consent. The University of Melbourne’s Human Research Ethics Committee approval (#1441865) for collection of supragingival dental plaque for microbial analysis was obtained. With the assistance of a sterile ultrasonic scaler supragingival plaque from the surface of canine mandible teeth was collected and transferred to a tube containing sterile phosphate buffered saline (PBS; 10 mM Na_2_HPO_4_, 1.8 mM KHPO_4_, 137 mM NaCl, 2.7 mM KCl pH 7.4) and immediately transferred to the laboratory. Each plaque sample was 10-fold serially diluted in PBS and 100 μl from 10^-4^, 10^-5^ and 10^-6^ dilution tubes spread on NA plates (0.5 % peptone, 0.3 % beef extract, 0.5 % sodium chloride, and 1.5 % agar) and were incubated overnight at 37 °C. Morphologically different colonies were selected and patched to fresh NA plates supplemented with 2 μg/ml of chlorhexidine gluconate (Sigma Aldrich). Resistant colonies were selected for further analysis.

Pure cultures of each were sent to Macrogen Inc., (Korea) for DNA extraction and 16S rRNA coding gene sequence analysis using their standard pipeline (http://www.macrogen.com/eng/business/seq_16s_rRNA_sequencing.html). Briefly, colonies were picked with a sterilized toothpick and suspended in 0.5 ml of sterile saline in a 1.5 ml centrifuge tube. The sample was centrifuged for 10 min at 10,000 rpm. After removal of supernatant the pellet was suspended in 0.5 ml of Insta Gene Matrix (Bio-Rad, USA). The sample was then incubated at 56 °C for 30 min then heated at 100 °C for 10 min. One μl of this solution containing the template DNA was added to 20 μl of a PCR reaction solution containing primers 27 F, AGAGTTTGATC(A or C)TGGCTCAG and 1492R, CGGTTACCTTGTTACGACTT. Thirty-five amplification cycles were performed with the following specifications, 94 °C for 45 s, 55 °C for 60 s, and 72 °C for 60 s. *E. coli* genomic DNA was used as a positive amplification control. Amplicons of approximately 1400 base pairs were obtained and purified using the Montage PCR Clean up kit (Millipore). The purified PCR products were sequenced by using the nested oligonucleotides 518 F, CCAGCAGCCGCGGTAATACG and 800R, TACCAGGGTATCTAATCC. Sequencing was performed using the Big Dye terminator cycle sequencing kit (Applied BioSystems, USA) with products resolved on an Applied Biosystems model 3730XL automated DNA sequencing system (Applied BioSystems, USA).

### Antibacterial susceptibility pattern of dental isolates

The antibiotic sensitivity profile of the selected oral bacteria was determined by the Kirby-Bauer Method of disk diffusion [54]. Species were grown overnight in nutrient broth then diluted to optical density (OD) at 600 nm of 0.02 in water. The diluted cultures were then spread plated using a sterile swab onto the surface of Mueller-Hinton agar plates. Antibacterial discs impregnated with vancomycin (30 μg), ampicillin (10 μg), kanamycin (30 μg), gentamicin (10 μg), tetracycline (30 μg), erythromycin (15 μg), and chloramphenicol (30 μg) were placed on the surface of agar. Plates were incubated at 37 °C for 24 h and zone of growth inhibition measured. *Escherichia coli* ATCC 25922 and *Staphylococcus aureus* ATCC 25923 were used as reference Gram-negative and Gram-positive antimicrobial-sensitive strains respectively [55].

### Microtiter biofilm detection assay

Biofilm formation in the presence of CHX was determined as previously described with some modification [56]. All the dental isolates were grown in 200 μl of nutrient broth in polystyrene 96-well microtiter plates for 3 days at 37 °C. Each well contained serially diluted CHX (1.9, 3.8, 7.6, 15.5, 32.0 62.5 and 125 μg/ml). Growth medium was removed and the plate was washed twice by submerging it in deionized water. Each well was treated with 0.2 ml absolute ethanol for 20 min to fix the residual bacteria. The plates were emptied, air dried and the adherent biofilm was stained for 10 min with 0.2 ml of 2.5 % (w/v) crystal violet (CV) and air dried. The plates were washed with distilled water thrice to rinse off excess stain. For each well, 160 μl of 33 % (v/v) glacial acetic acid was used to dissolve the dye bound to adherent cells and the solution transferred to a new microtiter plate. The OD of each well was measured at 570 nm using a Multiskan Ascent microtiter plate reader (Thermo Fisher Scientific). Three separate experiments were performed for each studied condition.

### Minimum inhibitory concentration (MIC) determination

The CHX MIC of isolates were assayed as previously described with some modifications [57]. Briefly, CHX from three different sources: chlorhexidine gluconate (Sigma Aldrich), Dentalife antiseptic with 0.2 % w/v chlorhexidine gluconate active ingredient; (Dentalife Australia, Ringwood Victoria, Australia) and Savacol mouthwash, also with 0.2 % chlorhexidine gluconate w/v active ingredient (Colgate) were prepared as stock solutions of 1000 μg/ml by dilution with sterile distilled water. Further dilutions were made from the stock using 1× Mueller Hinton Broth (MHB) to give 100 μl final volume in the wells of a sterile polystyrene microtiter plate. The bacterial inoculum of 3 × 10^4^ colony forming units (CFU) in 100 μl volume was added to each well and plates were incubated overnight at 37 °C. For each test plate, two controls were included, one was a sterility control (medium alone) and the other was a growth control (medium and bacterial inoculum without CHX). The sterility control well was used as a blank with OD values subtracted from the test control well OD values.

### Chlorhexidine mouthwash time exposure survival profile of plaque bacteria

Isolates were grown in a 96-well microtiter polystyrene plate with 180 μl of sterile MHB dispensed in each well and 20 μl of bacterial culture (OD 600 nm 0.1) added. Plates were incubated at 37 °C for 3 days. After 3 days, plates were washed twice with 1 × MHB media to remove the planktonic cells. Using a multichannel pipette the cells remaining adhered to wall of each well were exposed one row at a time to the undiluted CHX mouthwash for different time intervals (5, 10, 20, 30, 40, and 60 s). At the end of the time interval the CHX was aspirated and the wells washed with 200 μl of 1 × MHB. After washing, 200 μl of MHB was again added to each well and the plate incubated overnight at 37 °C. The planktonic culture was aspirated to a fresh plate and the OD of the culture was measured at 600 nm using a Multiskan Ascent microtiter plate reader. Two controls (sterility control and growth control) were also included for each test plate in three biological replicates.

### Transcription response of *C. indologenes* to chlorhexidine

*C. indologenes* was grown overnight in nutrient broth then inoculated at 1/100 volume into 50 ml of fresh media and incubated at 37 °C with shaking at 200 rpm. After 4 h 2 × 10 ml aliquots were removed with one aliquot transferred to vial containing chlorhexidine to give 16 μg/ml final concentration. Incubation was continued for 1 h, after which cells were harvested in 1 ml aliquots. To harvest, the aliquots were centrifuged in a microcentrifuge at 8000 × *g* for 2 min at 4 °C, the supernatants were removed and the pellets snap frozen in liquid nitrogen before storage at -80 °C. Four separate experiments were performed.

### RNA isolation and cDNA synthesis

RNA was isolated using Trizol (Invitrogen) with the addition of 1.4 ml of Trizol directly to the frozen cell pellet. After gentle mixing the sample was transferred to a 2 ml tube containing 0.1 mm silica spheres (MP Bio Matrix B, pre chilled at -20 °C). The sample was homogenised using a Precellys instrument (Bertin Technologies) with two cycles of 600 rpm for 23 s, with 5 s between cycles. The samples were snap-chilled on ice for 3 min then incubated for 5 min at room temperature. The beads were pelleted by centrifugation (12,000 × *g*) for 5 min at 4 °C and the supernatant removed to a fresh tube. Chloroform (280 μl) was added to the supernatant and the tube shaken vigorously for 15 s by hand. After incubation at room temperature for 3 min the sample was centrifuged at 16,000 × *g* for 10 min at 4 °C and the upper colourless phase transferred to a fresh tube. For RNA precipitation, 0.7 ml of isopropanol (100 %) was added and the sample incubated at -80 °C for 30 min. The sample was centrifuged at 16,000 × *g* for 15 min at 4 °C and the supernatant removed. To wash the pellet 1.4 ml of 75 % v/v ethanol was added, the tube was briefly vortexed then centrifuged at 16,000 × *g* for 10 min at 4 °C. After centrifugation the wash solution was discarded and the RNA pellet was air dried for 15 min prior to suspension in 60 μl of RNase free water. Contaminating DNA was removed using Turbo DNA Free following manufacturer protocol (Applied Biosciences Inc.). Total RNA was quantified using micro spectrometry (Nano-Drop Technologies, Inc.). Oligonucleotide primers Cep2 FP (AACCGTTCCTTTTGTCAGCC) and Cep2 RP (CAGCGAGTTGTGCCTGTAAG) were designed using Primer-BLAST [58] to produce a 158 base pair amplicon from the *C. indologenes* gene of locus CIN01S_RS05745. Removal of DNA was confirmed by PCR with Cep2 FP and Cep2 RP, with genomic DNA as positive control.

Synthesis of cDNA was performed using the iScript Reverse transcription kit (Bio-Rad) with 1 μg of total RNA per reaction set up according to manufacturer instructions. Complete reaction mix was incubated in a thermal cycler with reaction conditions of 25 °C for 5 min, 30 min at 42 °C and at 85 °C for 5 min. The cDNA was stored at -80 °C.

### Real-Time PCR conditions

All reactions were performed in triplicate 10 μl volumes using Universal SYBR Green Supermix (Bio-Rad). Master mixes were prepared for each 10 μl final reaction volume that include 2 μl of cDNA, 0.25 μl of each primer Cep2 FP and Cep2 RP, 5 μl of 2× Universal SYBR Green Supermix and 3 μl of RNase free water. Serial diluted genomic DNA served as reaction controls. PCR was conducted on a QuantStudio™ 7 Flex Real-Time PCR System (Applied Biosystems). Reaction conditions were: enzyme activation and DNA denaturation at 98 °C for 3 min followed by 40 cycles of 98 °C (denaturation) for 15 s and at 50 °C for 30 s (annealing and extension). The melt curve analysis was done using instrument default of 65 °C-95 °C for 5 s and 0.5 °C temperature increments. QuantStudio™ 7 Flex software generated the cycle threshold value (C_q_).

## Abbreviations

Ap: Ampicillin
CHX: Chlorhexidine
Cm: Chloramphenicol
Cq: Quantitative cycle
CV: Crystal violet
Em: Erythromycin
Gm: Gentamicin
Km: Kanamycin
MIC: Minimum inhibitory concentration
NA: Nutrient agar
OD: Optical density
PBS: Phosphate buffered saline
PCR: Polymerase chain reaction
Tc: Tetracycline
Vm: Vancomycin

## References

[1] Petersen PE, Bourgeois D, Ogawa H, Estupinan-Day S, Ndiaye C (2005). The global burden of oral diseases and risks to oral health. Bull World Health Organ.

[2] Seymour G, Ford P, Cullinan M, Leishman S, Yamazaki K (2007). Relationship between periodontal infections and systemic disease. Clin Microbiol Infect.

[3] Jenkinson HF, Lamont RJ (2005). Oral microbial communities in sickness and in health. Trends Microbiol.

[4] Roberts AP, Mullany P (2010). Oral biofilms: a reservoir of transferable, bacterial, antimicrobial resistance. Expert Rev Anti Infect Ther.

[5] Høiby N, Bjarnsholt T, Givskov M, Molin S, Ciofu O (2010). Antibiotic resistance of bacterial biofilms. Int J Antimicrob Agents.

[6] Gunsolley JC (2006). A meta-analysis of six-month studies of antiplaque and antigingivitis agents. J Am Dent Assoc.

[7] Haps S, Slot D, Berchier C, Van der Weijden G (2008). The effect of cetylpyridinium chloride‐containing mouth rinses as adjuncts to toothbrushing on plaque and parameters of gingival inflammation: a systematic review. Int J Dent Hyg.

[8] Stoeken JE, Paraskevas S, Van Der Weijden GA (2007). The long-term effect of a mouthrinse containing essential oils on dental plaque and gingivitis: a systematic review. J Periodontol.

[9] Albandar JM (2005). Epidemiology and risk factors of periodontal diseases. Dent Clin North Am.

[10] Barnett ML (2003). The role of therapeutic antimicrobial mouthrinses in clinical practice: control of supragingival plaque and gingivitis. J Am Dent Assoc.

[11] Löe H (2000). Oral hygiene in the prevention of caries and periodontal disease. Int Dent J.

[12] Pitten F, Splieth C, Kramer A (2000). Prophylactic and therapeutic application of antimicrobial agents in the oral cavity. Pharmazie.

[13] Denton GW (1991). Chlorhexidine. Disinfection, sterilization and preservation.

[14] Lang N, Brecx MC (1986). Chlorhexidine digluconate–an agent for chemical plaque control and prevention of gingival inflammation. J Periodontal Res.

[15] Gunsolley JC (2010). Clinical efficacy of antimicrobial mouthrinses. J Dent.

[16] Löe H, Rindom Schiøtt C (1970). The effect of mouthrinses and topical application of chlorhexidine on the development of dental plaque and gingivitis in man. J Periodontal Res.

[17] Block C, Furman M (2002). Association between intensity of chlorhexidine use and micro-organisms of reduced susceptibility in a hospital environment. J Hosp Infect.

[18] Morrissey I, Oggioni MR, Knight D, Curiao T, Coque T, Kalkanci A, Martinez JL, Consortium B (2014). Evaluation of epidemiological cut-off values indicates that biocide resistant subpopulations are uncommon in natural isolates of clinically-relevant microorganisms. PLoS One.

[19] Karpiński TM, Szkaradkiewicz AK (2015). Chlorhexidine – pharmaco-biological activity and application. Eur Rev Med Pharmacol Sci.

[20] Martin JF, Liras P (1989). Organization and expression of genes involved in the biosynthesis of antibiotics and other secondary metabolites. Annu Rev Microbiol.

[21] Webber MA, Whitehead RN, Mount M, Loman NJ, Pallen MJ, Piddock LJ (2015). Parallel evolutionary pathways to antibiotic resistance selected by biocide exposure. J Antimicrob Chemother.

[22] Giuliano CA, Rybak MJ (2015). Efficacy of triclosan as an antimicrobial hand soap and its potential impact on antimicrobial resistance: a focused review. Pharmacotherapy.

[23] Davies J (1994). Inactivation of antibiotics and the dissemination of resistance genes. Science.

[24] Qamar FN, Azmatullah A, Kazi AM, Khan E, Zaidi AKM (2014). A three-year review of antimicrobial resistance of *Salmonella enterica* serovars Typhi and Paratyphi A in Pakistan. J Infect Dev Ctries.

[25] Ayaz A, Hasan Z, Jafri S, Inayat R, Mangi R, Channa AA, Malik FR, Ali A, Rafiq Y, Hasan R (2012). Characterizing *Mycobacterium tuberculosis* isolates from Karachi, Pakistan: drug resistance and genotypes. Int J Infect Dis.

[26] Idrees F, Jabeen K, Khan MS, Zafar A (2009). Antimicrobial resistance profile of methicillin resistant *Staphylococcal aureus* from skin and soft tissue isolates. J Pak Med Assoc.

[27] Lin Y-T, Jeng Y-Y, Lin M-L, Yu K-W, Wang F-D, Liu C-Y (2010). Clinical and microbiological characteristics of *Chryseobacterium indologenes* bacteremia. J Microbiol Immunol Infect.

[28] Li X-Z, Plésiat P, Nikaido H (2015). The challenge of efflux-mediated antibiotic resistance in Gram-negative bacteria. Clin Microbiol Rev.

[29] Srinivasan VB, Rajamohan G (2013). KpnEF, a new member of the *Klebsiella pneumoniae* cell envelope stress response regulon, is an SMR-type efflux pump involved in broad-spectrum antimicrobial resistance. Antimicrob Agents Chemother.

[30] Baehni P, Takeuchi Y (2003). Anti‐plaque agents in the prevention of biofilm associated oral diseases. Oral Dis.

[31] Affairs ADACoS (1997). Antibiotic use in dentistry. J Am Dent Assoc.

[32] Michel C, Matte-Tailliez O, Kerouault B, Bernardet J-F (2005). Resistance pattern and assessment of phenicol agents’ minimum inhibitory concentration in multiple drug resistant *Chryseobacterium* isolates from fish and aquatic habitats. J Appl Microbiol.

[33] Kämpfer P, Chandel K, Prasad G, Shouche Y, Veer V (2010). *Chryseobacterium culicis* sp. nov., isolated from the midgut of the mosquito *Culex quinquefasciatus*. Int J Syst Evol Microbiol.

[34] Aydin-Teke T, Tanir G, Bayhan G, Metin O, Oz N (2014). Erythema nodosum in children: evaluation of 39 patients. Turk J Pediatr.

[35] Chen F-L, Wang G-C, Teng S-O, Ou T-Y, Yu F-L, Lee W-S (2013). Clinical and epidemiological features of *Chryseobacterium indologenes* infections: analysis of 215 cases. J Microbiol Immunol Infect.

[36] Hoffmann H, Stindl S, Stumpf A, Mehlen A, Monget D, Heesemann J, Schleifer KH, Roggenkamp A (2005). Description of *Enterobacter ludwigii* sp. nov., a novel *Enterobacter* species of clinical relevance. Syst Appl Microbiol.

[37] Mezzatesta ML, Gona F, Stefani S (2012). *Enterobacter cloacae* complex: clinical impact and emerging antibiotic resistance. Future Microbiol.

[38] Gales A, Jones R, Forward K, Linares J, Sader H, Verhoef J (2001). Emerging importance of multidrug-resistant *Acinetobacter species* and *Stenotrophomonas maltophilia* as pathogens in seriously ill patients: geographic patterns, epidemiological features, and trends in the SENTRY Antimicrobial Surveillance Program (1997–1999). Clin Infect Dis.

[39] Azarpazhooh A, Leake JL (2006). Systematic review of the association between respiratory diseases and oral health. J Periodontol.

[40] Gomes-Filho IS, Passos JS, da Cruz SS (2010). Respiratory disease and the role of oral bacteria. J Oral Microbiol.

[41] Grap MJ, Munro CL, Hamilton VA, Elswick RK, Sessler CN, Ward KR (2011). Early, single chlorhexidine application reduces ventilator-associated pneumonia in trauma patients. Heart Lung.

[42] Siempos II, Falagas ME (2007). Oral decontamination with chlorhexidine reduces the incidence of nosocomial pneumonia. Crit Care.

[43] Celakovsky P, Kalfert D, Smatanova K, Tucek L, Cermakova E, Mejzlik J, Kotulek M, Vrbacky A, Matousek P, Stanikova L (2015). Bacteriology of deep neck infections: analysis of 634 patients. Aust Dent J.

[44] Maripandi A, Kumar A, Al Salamah AA (2011). Prevalence of dental caries bacterial pathogens and evaluation of inhibitory concentration effect on different tooth pastes against *Streptococcus* spp. Afr J Microbiol Res.

[45] Koljalg S, Naaber P, Mikelsaar M (2002). Antibiotic resistance as an indicator of bacterial chlorhexidine susceptibility. J Hosp Infect.

[46] Blair JM, Richmond GE, Piddock LJ (2014). Multidrug efflux pumps in Gram-negative bacteria and their role in antibiotic resistance. Future Microbiol.

[47] Rajamohan G, Srinivasan VB, Gebreyes WA (2010). Novel role of *Acinetobacter baumannii* RND efflux transporters in mediating decreased susceptibility to biocides. J Antimicrob Chemother.

[48] Hassan KA, Jackson SM, Penesyan A, Patching SG, Tetu SG, Eijkelkamp BA, Brown MH, Henderson PJF, Paulsen IT (2013). Transcriptomic and biochemical analyses identify a family of chlorhexidine efflux proteins. Proc Natl Acad Sci U S A.

[49] Shen Y, Stojicic S, Haapasalo M (2011). Antimicrobial efficacy of chlorhexidine against bacteria in biofilms at different stages of development. J Endod.

[50] Bonesvoll P, Gjermo P (1978). A comparison between chlorhexidine and some quaternary ammonium compounds with regard to retention, salivary concentration and plaque-inhibiting effect in the human mouth after mouth rinses. Arch Oral Biol.

[51] Wilson M (1996). Susceptibility of oral bacterial biofilms to antimicrobial agents. J Med Microbiol.

[52] Westergren G, Emilson C-G (1980). In vitro development of chlorhexidine resistance in *Streptococcus sanguis* and its transmissibility by genetic transformation. Eur J Oral Sci.

[53] Sreenivasan P, Gaffar A (2002). Antiplaque biocides and bacterial resistance: a review. J Clin Periodontol.

[54] Baur A, Kirby W, Sherris J, Turch M (1966). Antibiotic susceptibility testing by a standardized single disc method. Am J Clin Pathol.

[55] Wayne P. Performance standards for antimicrobial susceptibility testing. Ninth informational supplement NCCLS document M100-S9. National Committee for Clinical Laboratory Standards. 2008;120–6.

[56] Chen C, Liao X, Jiang H, Zhu H, Yue L, Li S, Fang B, Liu Y (2010). Characteristics of *Escherichia coli* biofilm production, genetic typing, drug resistance pattern and gene expression under aminoglycoside pressures. Environ Toxicol Pharmacol.

[57] Wiegand I, Hilpert K, Hancock RE (2008). Agar and broth dilution methods to determine the minimal inhibitory concentration (MIC) of antimicrobial substances. Nat Protoc.

[58] Ye J, Coulouris G, Zaretskaya I, Cutcutache I, Rozen S, Madden T (2012). Primer-BLAST: A tool to design target-specific primers for polymerase chain reaction. BMC Bioinformatics.

